# How survival curves affect populations’ vulnerability to climate change

**DOI:** 10.1371/journal.pone.0203124

**Published:** 2018-09-06

**Authors:** John M. Halley, Kyle S. Van Houtan, Nate Mantua

**Affiliations:** 1 Department of Biological Applications and Technology, School of Health Sciences, University of Ioannina, Ioannina, Greece; 2 Monterey Bay Aquarium, Monterey, CA, United States of America; 3 Nicholas School of the Environment and Earth Sciences, Duke University, Durham, North Carolina, United States of America; 4 NOAA Fisheries, Southwest Fisheries Science Center, Santa Cruz, California, United States of America; Fred Hutchinson Cancer Research Center, UNITED STATES

## Abstract

Human activities are exposing organisms not only to direct threats (e.g. habitat loss) but also to indirect environmental pressures such as climate change, which involves not just directional global warming but also increasing climatic variability. Such changes will impact whole communities of organisms and the possible effects on population dynamics have raised concerns about increased extinction rates. Conservation-minded approaches to extinction risk vary from range shifts predicted by climate envelope models with no population dynamics to population viability analyses that ignore environmental variability altogether. Our modelling study shows that these extremes are modelling responses to a spectrum of environmental sensitivity that organisms may exhibit. We show how the survival curve plays a major role in how environmental variability leads to population fluctuations. While it is often supposed that low-fecundity organisms (those with high parental investment) will be the most vulnerable to climate change, it is those with high fecundity (low parental investment) that are likely to be more sensitive to such changes. We also find that abundance variations in high fecundity populations is driven primarily by fluctuations in the survival of early life stages, the more so if those environmental changes are autocorrelated in time. We show which types of conservation actions are most appropriate for a number of real populations. While the most effective conservation actions for organisms of low fecundity is to avoid killing them, for populations with high fecundity (and low parental investment), our study suggests conservation should focus more on protecting early life stages from hostile environments.

## Introduction

Currently expected climatic change involves not only higher average temperatures but may also involve increasing variability [1, 2]. Future climate change [3, 4] will impact whole communities, not just single populations, which has raised concerns about increased extinction rates [5]. An understanding of how increases in climate variance will affect population variability is critical for managing species protected through statutory processes, such as the U.S. Endangered Species Act, to promote their long-term persistence [6, 7].

Much of the current effort to forecast the ecological effects of climate change is habitat or niche based. A popular approach is to consider climate envelopes and how their changes will affect species distributions. Within these envelopes, populations of organisms operate in their ecological niche, yet outside of these envelopes they fail to thrive [8]. As a result, there are many studies on shifts in geographic distributions, poleward or upslope migrations, phenological mismatches, and the collective effects of these shifts to trophic dynamics [9–13]. In the above approaches, population dynamics and organism life history traits tend to be ignored, although recently there has been a drive to include more demography into species distribution models [14–18]. By contrast, in conservation biology population dynamics usually plays a major role. Classic textbooks and various packages are available to explore the dynamics of specific populations with and without age structure [19, 20]. These approaches tend to emphasize counts of breeding adults (often of birds and mammals) while diminishing that of environmental variability. In fisheries science, explicit links between demographics and environmental factors are more commonly employed and environmental variability is understood to play a significant role in population dynamics, primarily through the well-documented mechanisms of somatic growth and recruitment of juveniles into the population [21–24]. Future projections of the impact of climate change on fisheries, as a result, often incorporate climatic controls on ecosystem productivity and its impact to juvenile recruitment to forecast population changes [25, 26].

Thus, we see several apparently contradictory approaches to extinction risk in the context of climatic change. These range from climate-change studies that regard the environment as paramount, to PVA studies in conservation biology that tend to regard changes in the environment as relatively unimportant. All of these are logical approaches in the appropriate limiting cases. Since climatic change will impact entire communities, ultimately a more unified approach is needed.

Two specific examples have motivated our interest in this question. The first is a recent debate about protected species management, on the influences of climate variability to marine turtle populations. Underscoring the role of climate to juvenile recruitment, Van Houtan & Halley [27] predicted 25 time series of loggerhead sea turtle (*Caretta caretta*) nesting populations in the Atlantic and Pacific Oceans using hatch year and breeding year climate indices. In response, Arendt et al. [28] argued that loggerhead nesting numbers in the Atlantic could be modelled using only contemporary climate indices, thus giving negligible environmental influence to juvenile recruitment. Then, Ascani et al. [29] demonstrated ocean circulation dynamics vary decadally and have dramatic influences on ecosystem productivity, neonate survival, and likely explain observed loggerhead nesting trends. These issues are particularly relevant for protected sea turtle populations, where quantifying the relative influence of bottom-up (ecosystem) and top-down (e.g., fisheries bycatch) forces informs decisions about the effectiveness of various conservation and management strategies [30].

The second concerns the relative influence of harvesting and environmental disturbance on the sustainability of fungi. Wild-mushroom collecting is an important activity in many countries and sustainability is obviously an important consideration. For this reason, often there are calls to limit the collection or harvesting of wild mushrooms. However, recent work investigating the effects of sustained harvesting unexpectedly found that the intensity of harvesting itself had little effect but that harvest-related damage to the immediate environment could have serious effects on subsequent yields [31, 32]. This suggests that removing individuals causes a smaller impact on fungi production/availability than does damage to the surrounding environment. Such a result goes strongly against the intuition of most conservation biologists. Nevertheless, these results have been reproduced [31, 32].

In conservation terms, what is the difference between organisms like seals on the one hand and organisms like fungi on the other, and in which group do sea turtles belong? In both cases, the underlying issue is whether climate change can be expected to be the main influence and at which stage of the life-cycle is this influence greatest. Is it the extreme fecundity of fungi (a single fruiting body may release billions or even trillions of spores) that makes them apparently insensitive to removal of individuals, but sensitive to habitat changes? It is our contention that the crucial difference lies in each organisms’ strategy as embodied in their survival curve, where there is a symmetry between mortality and fecundity (high fecundity implies high mortality and vice versa). It is this symmetry in birth rates and death rates that determines the kind of analysis required and the kind of management strategy for conservation that should be pursued. We will argue that organisms with high fecundity have a fundamentally different response from organisms such as seals and elephants and hence different conservation requirements.

Much has been said about life history and how it determines organisms’ response to environmental variation. There is a vast corpus of ecological theory dedicated to this issue. The organisms’ response depends on many things: longevity [33], generation time [34], demographic dispersion [35], demographic versus environmental noise amplitude [33, 36], noise correlation [37], non-stationary noise [38] and many other things, including combinations of the above. Attempts to model the effect of climatic changes often focus on specific types, such mammals or birds [34, 39–41] or plant populations regulated by density dependence [14–16] but insights gained can be extended to more general situations. Nevertheless, in this great wealth of literature, we found relatively little on the specific role of fecundity. In fisheries science, the issue has been recognized as important in the “recruitment problem” (i.e. why is recruitment so variable?) leading to possible problems in the context of conservation [42–44]. However, the large-scale studies such as those by Mertz & Myer [43] or Rickman et al [45] tend to consider commercially important stock species which fall disproportionately on the high fecundity end in the range 10^3^ to 10^6^ (offspring per female over the individual’s lifetime?) Despite this wide range, the absence of low fecundities in the range 1 to 10^3^ is a major limitation since much of the crucial differences in strategy fall within this range. The treatment of this parameter in the terrestrial literature is even more cursory. This shows that a greater focus on fecundity is needed.

**Fig 1:**
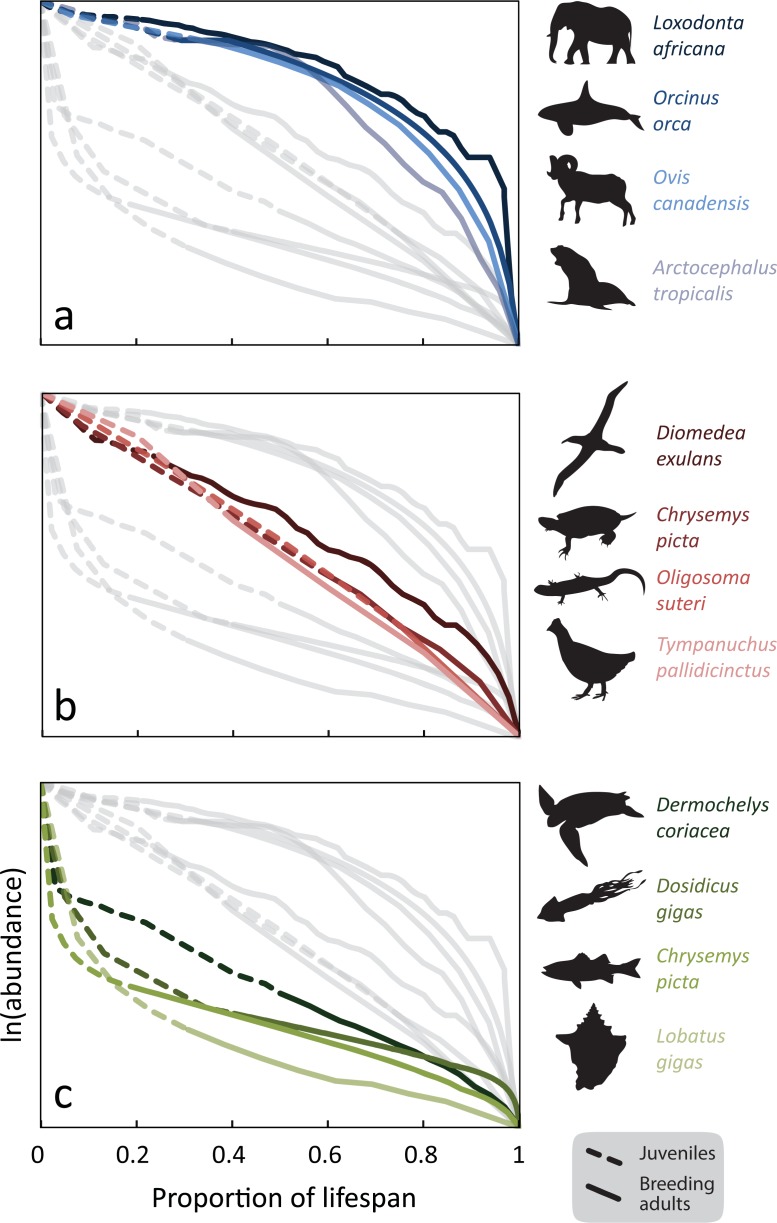
Three life history types expressed in terms of abundance and survival over time. Type I populations (top panel) that are characterized by low fecundity, high survival, longer life span, with reproductive senescence. Type III (bottom panel) populations are characterized by high fecundity, low juvenile survival, with increasing survival of later age classes. Type II populations (middle panel) are an intermediary form with relatively constant survival throughout all age classes. Dotted lines represent juvenile stages; solid lines begin at the earliest onset of breeding. Lines from bordering panels are retained as faint grey lines for reference. Both abundance and lifespan proportion are normalized and used to find the metric *A* of life-history type (see Appendix B in S1 File). The average inverse survival to maturity is for the three types are 1.5, 6.3 and 84,000 respectively).

This paper thus focuses on individual fecundity and the patterns of mortality prior to reproduction and how these determine the response of populations to environmental variability. We use the classical scheme introduced by Pearl [46] that distinguishes species according to their survival curve (Fig 1). These range from “Type I” where parents invest most of their reproductive energy in safeguarding offspring, so that mortality is low in the early stages of life through to “Type III” populations which invest most of the energy in maximizing fecundity but where immature individuals experience extreme mortality. “Type II” strategies lie intermediate, involving relatively constant mortality throughout life. By using a modelling approach, we show that as we move away from a Type I and towards Type III survival strategy, population variability increasingly reflects environmental variation, especially that in earlier life stages. [46–48].

Fig 1 illustrates these different strategies for a number of real organisms. In it we normalized both abundance and lifespan survival rates in order to compare different strategies employed in the lifetime survival profiles for a variety of organisms. On the vertical axis, data range from the initial maximum at *N*_0_ (cohort production) down to the last individual, scaled by *n*_0_ = ln(*N*_0_). The horizontal axis is normalized relative to the maximum lifespan. For Type I species we observe a low mortality rate for early life stages followed by a stable mortality rate plateau until senescence when survival drops rapidly (Fig 1A). For type II, there is a more or less exponential decline in numbers. Type III species suffer rapid loss in early life followed by slower loss as adults. In this evolutionary wager, high mortality rates in the early life-stages are offset by the sheer numbers of offspring. An additional evolutionary payoff is that natural selection acts quickly. (Note that the evolutionary progression runs from Type III to Type I with the development of traits such as endothermy, live birth, parental care and social behaviour.) Type-III has sometimes been referred to an *r*-strategy (referring to how the intrinsic per-capita rate of increase, *r*, is maximized) in contrast to the *K*-strategy of Type I. However, these terms have fallen under scrutiny (e.g., [49]) and are no longer popular. All data are from published studies [50–64]. One of the aims of this figure is to compare a large sample of such strategies, so as to span the evolutionary tree. However, it is obvious that there exist more extreme Type III species. Fungi have fecundity that may be measured in billions or even trillions [65, 66].

The survival strategy can be quantified through various metrics. Often these may involve three or more parameters that often target specific taxonomic groups such as plants [67] or fish [68]. One-parameter metrics are much cruder but may capture common patterns across taxonomic groups. Such metrics include, for example, the inverse proportion of individuals that reach the reproductive phase *S*_m_^-1^, or the mortality rate in the first year, or *μ*_0_, or adult fecundity, or steepness. A measure using the relative area subtended by the survival curve above or below the diagonal in Fig 1 is described in Appendix B in S1 File. In this paper we will be concentrating mainly on the mortality rate *μ*_0_ and on adult fecundity, which, as we shall argue, are closely related to one another. Fecundity ranges a million-fold in nature across the organisms we consider. We show that survival curves affect the gains of different conservation actions and so we argue that it should be a more central feature of practical and urgent conservation decisions for commercially-exploited and protected species.

We assume there are no significant density-dependent compensatory mechanisms in operation in the populations considered. Although these effects should be an important part of this story [69], we believe their role is still not always understood [70] and is likely to differ across the wide range of organisms we consider, so we defer a discussion of their effects to a later paper. Here, we will use two idealized density-independent stochastic population models. The first model is used to derive explicit results for the response to influence from environmental variability. We show how the dynamics of populations with Type III strategies should have stronger influence from environmental variability. The second model generalizes the first model by allowing age-structured survival and fecundity. This Leslie matrix model is parameterized with demographic data for various species. We then use simulations that confirm the results of model-1, showing that population variability due to environmental changes increases as Type III strategies that are more extreme. We further consider the sensitivity of extinction risk to varying autocorrelation in environmental forcing. We show a practical application of these analyses through weighing the benefits of a variety of conservation actions across demographic units of populations with Type I, Type II, and Type III life history strategies. Although our model ignores many important features of population dynamics such as spatial effects and density dependence, which will need to be addressed if we wish to apply our findings to conservation decisions, we believe our framework brings new insights about the interaction between life history strategies and different types of environmental stochasticity including climatic changes for a large range of organisms.

## Methods

Although the two extremes need not differ in terms of the expected mean reproductive successes, there is a great difference in terms of how fluctuations of the environment are reflected as population responses. Since the high mortality rate for Type III species happens *before* breeding, it means that the numbers breeding are strongly affected by juvenile mortality as well as adult fecundity changes.

**Fig 2:**
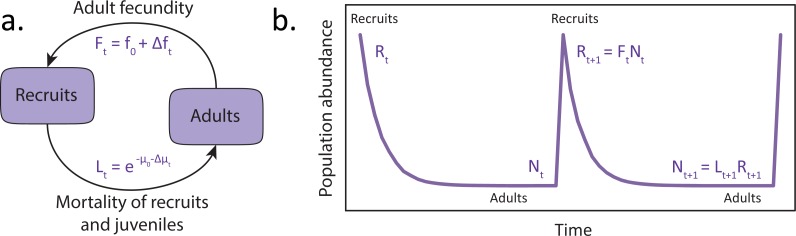
(a) Simplified schematic of the life cycle of an organism described by Model 1. The environmental fluctuations in fecundity may be described by Eq (A7), a constant plus or minus *an environmental perturbation*. However, for survival the environment intrudes through the probability of a given individual dying within a given time-frame—the environment intrudes in a multiplicative way. (b) Dynamics of the total population of the organism. In general, the depth of oscillation will be greater for Type III organisms.

We develop models to predict the variability of populations of specific life-history types responding to environmental noise. We describe population dynamics through two simple models. Fig 2A describes Model 1, which we use to illustrate the basic concepts. In this model, organisms reach adulthood relatively late in life, reproduce, and immediately die, so the maximum lifetime, *T*, is also the generation time and the time to maturity. Each generation suffers attrition defined by *L*_*t*_, the survival to reproductive maturity. The numbers jump up again when the next generation is born. The difference in the numbers of young recruits versus adults is a measure of the degree to which a species’ strategy is Type III.

The dynamics of the total population in Model 1 are shown in Fig 2B and can be simply represented by the relation:

$$N_{t+1}=R_{t}N_{t}=f_{t}\cdot L_{t}\cdot N_{t}$$ (1)

Here *f* is the fecundity and the subscripts signify the time dependence. Equations of this type are extremely general and can describe a wide variety of systems. They do not require the diffusion approximation [36], which is needed when using stochastic differential equations [71], and so can be applied to Type III as well as Type I organisms. When *R* is stochastic and density dependent, they require technical approaches such as integral equations [36, 72]. Such models have been applied to various taxonomic groups including plants, insects [73] and amphibians [74]. We use the simpler density-independent form above because it is commonly used in simulating the probability of low population sizes and requires the fewest number of assumptions about parameters [75] as well as being much easier to analyze.

The coefficient of replacement (R) is the number of female offspring each female can expect to produce in her lifetime. When *R*>1 the population grows exponentially, if *R*<1 it declines exponentially, and if *R* = 1 the population is stable. This can be expressed as *R* = *fL*. The first factor *L* reflects infant and juvenile survival while the second, *f*, is the fecundity of the adult stages. Thus, the total replacement rate can be expressed as a product of the total survival, from the beginning to adulthood, times the total reproduction over all stages of maturity. In the absence of stochasticity, the mortality rate *μ*_0_ is related to *L* through the time *T* to maturity by *L* = exp(-*μ*_0_*T*). In the case where the species is neither growing nor declining from one generation to the next, *R* = 1, mortality and fecundity must thus have similar orders of magnitude (Fig 2) so:

$$\mu_{0}T\approx \ln(f)$$ (2)

Eq (2) illustrates an expected symmetry between the average per-capita fecundity and mortality but this does not hold true of variability about these average values. In the presence of environmental variability in both fecundity and mortality, the factors *f* and *L* become time-dependent. However, environmental stochasticity enters fecundity and mortality in different ways. Regarding fecundity, adult females prepare for reproduction by accumulating resources for reproduction each day. We assume that the number of eggs (or egg-equivalents) that can be stored per day is a physiological process that is modulated by an environmental variable. Since these egg-equivalents added per day do not depend strongly on the amount already accumulated, we can think of the environmental effect Δ*f*_*t*_ as a sum of Gaussian perturbations (see Eq (A7) in Appendix A in S1 File). This factor remains Gaussian, even when fecundity is very large. The mortality process is different. Each day, the numbers dying depend on an average mortality modulated by an environmental variable. But the daily number of individuals that die also depends on the population at the start of that day. As we move from one day to the next the process is multiplicative leading to a *lognormal* distribution [43, 76]. In the presence of environmental variability of both fecundity and mortality, Eq (1) becomes:

$$N_{t+1}=\left(f_{T}+\Delta f_{t}\right)\cdot L\cdot \exp\left[-\Delta \mu_{t}\right]\cdot N_{t}$$ (3)

See results (A2) and (A7) in Appendix A for details. Here *f*_*T*_ is the mean lifetime fecundity of an individual. The (Normal) random variables Δ*f*_*t*_ and Δ*μ*_*t*_ are the respective (Gaussian) environmental fluctuations on these parameters.

If we assume Eq (2) holds but with small perturbations around the equilibrium, we can find the equation for the time-dependent growth rate of the population (See Appendix B in S1 File). This gives an average growth rate of zero plus two noise terms, the first associated with fecundity and the second with survival, which leads to the following formula for the overall variance of the growth rate:

$$V_{r}=\left(V_{f}+\mu_{0}^{2}T^{2}V_{S}\right)\frac{v(T)}{T}=\left(V_{f}+{\left(\ln f\right)}^{2}V_{S}\right)\frac{v(T)}{T}$$ (4)

Here *V*_*f*_ and *V*_*S*_ are the variances of the daily *proportional* changes to *f* and to *μ* due to environmental fluctuations. *T* is the time to maturity and the function *v*(*T*) is a measure of the autocorrelation of environmental variability, assumed the same for both perturbations: *v*(*T*) is unity for uncorrelated variability and increases more strongly with *T* as the autocorrelation in variability increases. It is proportional to *T*^2^ for Brownian motion. Thus, for this simple model, provided *V*_*f*_ and *V*_*S*_ are comparable then the contribution to *V*_*r*_ from environmental fluctuations on the mortality rate is multiplied by the factor (ln*f*)^2^ and so increases as the fecundity increases. Further increases of fecundity towards more extreme Type III strategies cause the perturbation approximation [3] to break down because of the lognormal distribution that further accentuates the asymmetry.

In order to accommodate organisms with more complex life histories we used Model 2, which is the standard Leslie matrix model with *T*+1 ages, where *T* is the maximum longevity. Model-2 is a more realistic and general version of Model-1, since survival is not constrained to be constant and reproduction is not confined to the end of the cycle. The total population in the year *t* is given by:

$$N_{t}=\sum_{k=0}^{K}{\left(n_{k}\right)}_{t},$$ (5)

Here *n*_*k*_ is the population in age class *k*. Note that in this model as in Model 1 we can have non-integer population values. The age-structured population values are related by the standard equation for Leslie matrices:

$${\left(\begin{array}{l} n_{0}\\ n_{1}\\ n_{2}\\ \vdots \\ n_{T-1}\\ n_{T} \end{array}\right)}_{t+1}={\left(\begin{array}{llllll} f_{0,t} & f_{1,t} & f_{2,t} & \ldots & f_{T-1,t} & f_{T,t}\\ s_{0,t} & 0 & 0 & \ldots & 0 & 0\\ 0 & s_{1,t} & 0 & \ldots & 0 & 0\\ \vdots & \vdots & \vdots & \ddots & \vdots & \vdots \\ 0 & 0 & 0 & \ldots & 0 & 0\\ 0 & 0 & 0 & \ldots & s_{T-1,t} & 0 \end{array}\right)}_{t}{\left(\begin{array}{l} n_{0}\\ n_{1}\\ n_{2}\\ \vdots \\ n_{T-1}\\ n_{T} \end{array}\right)}_{t}$$ (6)

When environmental stochasticity is present, each parameter is affected equally. Noise enters the population dynamics through the survival coefficients, *s*_*kt*_ which are related to the mortality rates, *μ*_*k*_, as follows: and takes the form:

$$s_{kt}=\exp[-\mu_{k}-\mu_{k}\varepsilon_{t}],\;0\leq k\leq K$$ (7)

The steady-state survival *s*_*k*_ can be associated with the mortality rate: *s*_*k*_ = exp(-*μ*_*k*_). In the random factor, *ε*_*t*_ is in general a Gaussian autoregressive term (first-order) of mean zero and variance *σ*^2^, namely:

$$\varepsilon_{t+1}=\rho \varepsilon_{t}+(1-\rho^{2})\delta_{t},\;\delta_{t}∼N(0,\sigma^{2})$$ (8)

The first age of reproduction *k*_min_ and the last age of reproduction *k*_max_ are based on real measurements but we assume that average fecundity *f*_0_ is the same for all reproductive ages. Thus:

$$\begin{array}{c} f_{kt}=f_{0}+\Delta f_{t}\;\forall k_{\min}\leq k\leq k_{\max}\\ =0\;\mathrm{otherwise}. \end{array}$$ (9)

Model 1 could be thought of as a model for organisms that have either a single age-class with reproduction at the end of the cycle or one with many age classes all with the same survival. Model 2 is more realistic than Model 1, as it can accommodate overlapping generations and contains age-structured survival, but it still involves some simplifying assumptions. As well as ignoring density dependence we assume all age classes experience the same proportional perturbation in a given year, as implied by Eq (7). Koons *et al*. 2016 [38] present a very general framework for the analysis of systems of this kind but in this paper we will use a simulation approach described below and in Appendix C S1 File.

Simulations of Model 2 used survival rates estimated from natural populations. The precise value of fecundity is chosen so that the overall average growth rate is zero, the same approach as adopted elsewhere [77]. When environmental stochasticity is present, each parameter is affected equally. Noise, in general autocorrelated, enters the population dynamics through the mortality rate and fecundity, so that each survival term *s*_*k*_ is multiplied by a random factor (See Appendix A in S1 File). We used 50 age classes in total. In the case of organisms with an annual clock each class corresponds to a year. In model 2 organisms in their first year are referred to as “recruits” while all other pre-reproductive individuals are “juveniles”.

Environmental autocorrelation also affects population variability and can accentuate the effects of high mortality in early life stages. We use simulations of Model-2 to evaluate the combined effect of increasing mortality and environmental autocorrelation. For these simulations we chose different values of *s*_0_ and different values of the correlation coefficient *ρ* of the environmental variability. In each simulation the other survival coefficients *s*_*k*_ were those for the leatherback sea turtle. The fecundities *f*_*k*_ for the leatherback were also used, without environmental variability. In the presence of environmental variability all survival coefficients *s*_*k*_ were multiplied by the random variable exp(*ε*_*t*_) as in Eq (6). Here *σ*^2^, the variance in Eq (7), is chosen such that the standard deviation of *ε*_*t*_ is exactly 0.1 over the duration of the run.

We also investigate the responses of different kinds of populations to different conservation actions using Model 2. We define “conservation gain” based on average increases in annual growth rate (*r*) achieved by an action carried out annually. Examples of specific conservation actions might include saving 100 nests (for turtles), decreasing juvenile annual mortality by 0.01 or decreasing *μ*_0_ by 1% (see Table 1). Ideally, these would be standardized according to their cost of implementation but economic investigations are beyond the intended scope of this article. We standardize the levels of intervention so as to be equivalent to saving 100 nests for the Wandering Albatross (Type II). Saving 100 nests for the Wandering Albatross has a conservation gain of unity *G* = 1 when population is 1000. We adjusted the levels of all other interventions so as to yield this same value of *G* = 1 (with a population of 1000). In other words, the values of *x* in Table 1 were chosen so that, when each of the various actions is carried out for Wandering Albatross, the effect on *r* (in the absence of environmental stochasticity) is the same as the decrease in nest mortality achieved by saving 100 Wandering Albatross nests when the total population size is 1000. The model is set up so that the age-structure is stable and the growth rate zero before applying the conservation action. With intervention levels thus fixed, we ran 1000 simulations for each species at each intervention type for the same set of environmental stochasticity. Environmental stochasticity was fixed with *σ* = 0.1 with no autocorrelation. We then looked at what happened when the same actions were applied to other organisms with different strategies. We ran each model for 250 years and found the average *r* using linear regression for the last 150 years of each run. For the same conditions of environmental stochasticity, conservation gain was the difference in yearly growth rate (as a percentage) between the populations boosted by a conservation mechanism and those without a boost.

This model analysis is highly simplified. For example, our models do not consider evolutionary changes, spatial structure or density dependence. We also do not study a number of life-history traits such as semelparity, demographic dispersion, generation time or longevity. While all these and others may influence population dynamics, exploring these factors is beyond the intended scope of our analysis.

**Table 1:** Mean conservation gain, *G*, observed in five different species and life history types for nine different conservation actions. We define “conservation gain” as *G* = (*r-r*_0_)×200, where the model is set up so that growth rate is zero before applying either stochasticity or the conservation action. Then *r*_0_ is the growth rate with stochasticity but without the conservation action while *r* is the growth rate with both stochasticity and the conservation action. *N*_*a*_ for Action-9 denotes the adult population. All results are based on simulations using Model 2. Organisms in age-class *k* = 0 are referred to as “recruits”; other pre-reproductive individuals are “juveniles”. Larger sensitivities shown are in bold for emphasis. Species are DE = *Diomedea exulans* (albatross), OO = *Orchinus orca* (orca), DC = *Dermochelys coriacea* (leatherback sea turtle), CP = *Chrysemys picta* (Striped bass) and LG = *Lobatus gigas* (Queen conch).

Action		Effect on parameters	Size of action, x	*DE*	*OO*	*DC*	*CP*	*LG*
1	Increase recruit survival by amount *x*	*s*_0_ → *s*_0_ + *x*	0.100	1	0.75	**15.2**	**71.2**	**212.2**
2	Increase recruit survival by proportion *x*	*s*_0_ → *s*_0_(1+*x*)	0.115	1	0.22	1.1	1.6	3.5
3	Reduce recruit mortality by proportion *x*	1−*s*_0_ → (1−*s*_0_)(1−*x*)	0.743	1	0.16	**33.5**	**110.3**	**288.0**
4	Reduce mortality rate *μ*_0_ by proportion *x*	*μ*_0_ → *μ*_0_(1−*x*)	0.756	1	0.16	**27.3**	**86.2**	**223.6**
5	Increase all juveniles’ survival by *x*	*s*_*k*_ → *s*_*k*_ + *x*	0.005	1	**0.95**	1.2	1.3	3.4
6	Increase all juveniles’ survival by proportion *x*	*s*_*k*_ → *s*_*k*_(1+*x*)	0.006	1	**1.02**	1.0	0.9	0.7
7	Reduce all juveniles’ mortality by proportion *x*	1−*s*_*k*_ → (1−*s*_*k*_)(1−*x*)	0.066	1	0.11	3.1	5.1	29.7
8	Reduce *μ*_*k*_ for juveniles by x	*μ*_*k*_ → *μ*_*k*_(1−*x*)	0.069	1	0.11	2.9	4.0	12.7
9	Save/add *x* nests per year or equivalent	*f*_*k*_ → *f*_*k*_(1+*x*/*N*_*a*_)	100	1	**0.89**	1.1	0.2	0.3

## Results and discussion

Our results show that life history, specifically the survival curve and fecundity, plays a major role in the response of organisms to environmental change. Since the variability experienced by any species affects its vulnerability to extinction, survival curves may have a role to play in deciding conservation actions.

Model 1 yields a number of explicit theoretical results. Eq (4) shows that while the variance in growth rate responds proportionately to variability in fecundity (*f*), it is boosted by a factor *μ*_*T*_^2^ in its response to variability in the pre-reproductive mortality rate (*μ*). This factor is large (>>1) if fecundity is large, as is the case in Type III species. Thus, high fecundity populations are expected to have higher variability [45, 76]. This will play a role in the boom and bust cycles sardines and anchovies (e.g., [21]), where variability of mortality is high in the early stages, although nonlinear phenomena need to be included to understand fully what’s going on. This model also shows (via Eq (A16)) that stages having larger mortality contribute more variability to population fluctuations. Similarly, Eq (A17) shows that shorter-duration classes with similar mortality contribute more variability. This is consistent with the findings of others [33, 34] that increasing generation times lead to more stable populations.

**Fig 3:**
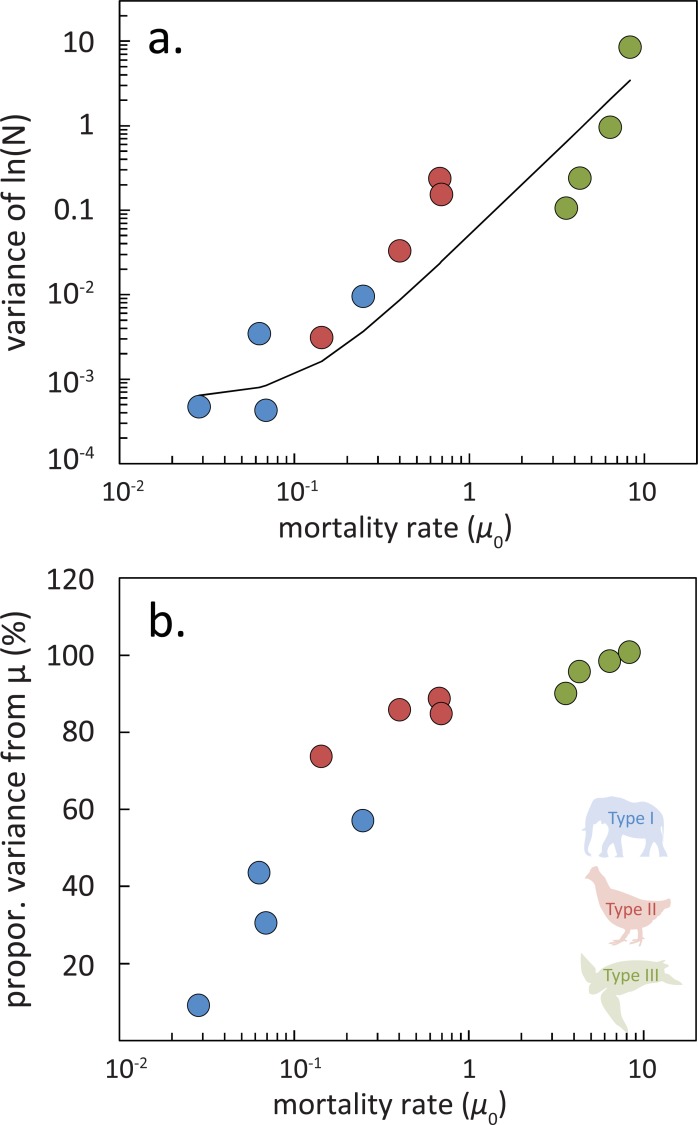
Sensitivity of variance in population (ln*N*_*t*_) to variability of the mortality rate *μ*_0_ for the youngest sub-adult class. We used simulations of Model 2 with noise (uncorrelated). (a) Total variance as a function of the value of mortality rate *μ*_0_ in the earliest stage. The distribution of strategy-types shows that Type III organisms have greater *μ*_0_ and this leads to a greater proportion of the overall variance in populations being dependent on the variability of this parameter. The distribution of the major strategy types shows that Type III organisms have greater *μ*_0_ and this leads to a greater overall variance in population growth rate. The curve corresponds to a best fit, after Eq (4), of the equation $V=a+b\mu_{0}^{2}$ based on Eq (4). (b) Proportion of variability explained by variations in pre-reproductive survival for different organisms (see Fig 1) as a function of *μ*_0_.

These patterns also hold for predictions made using the more realistic Model 2, which has age structure with all parameters subject to environmental stochasticity. Fig 3A shows how much of this stochasticity finds its way into population variance as a function of the mortality rate *μ*_0_ in the youngest sub-adult class. As *μ*_0_ increases so does the variance in population. Fig 3B shows how much of this variance is coming from the youngest class. As *μ*_0_ increases this proportion increases. Fig 3A and 3B demonstrate that Type III species have a greater sensitivity to environmental variability than Type I species, greater overall variance and are affected to a greater extent by survival variability. Type III species, with large reproduction and low parental input, are more responsive to environmental fluctuations affecting pre-reproductive stages. When we observe the trajectories of the adult populations in time, we expect the adult population size to show the greatest correlation with survival (and hence the environmental conditions prevailing) in the earliest phase of life. Type I and II species show a more even distribution across life stages.

**Fig 4:**
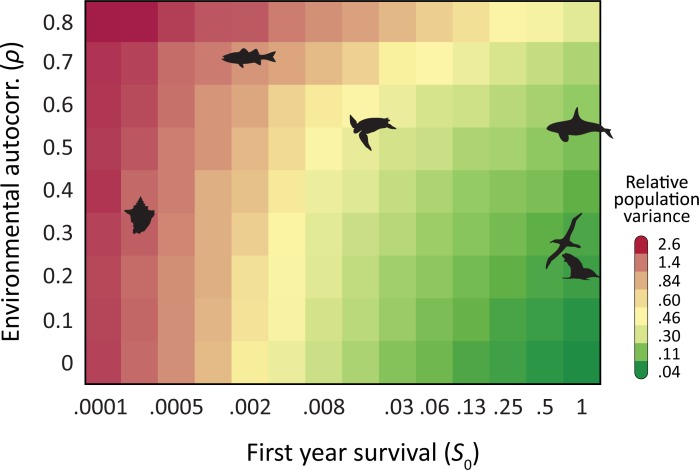
High mortality of early life stages and increased environmental autocorrelation have a combined effect that increases relative population variance. In order to study the effect of autocorrelation we find the variance of the log-population as a function of first year survival (*s*_0_) and autocorrelation coefficient *ρ* of environmental variability. Here the variance of *δ*_*t*_ is chosen such that the variance of *ε*_*t*_ is exactly 0.1 for the duration of model time. For each set of parameters, in 500 replicate simulations we ran a Leslie matrix model for 250 years of model time. For each replicate we found the variance of log-population and plot the average of this over 500 replicate simulations. We run these simulations across 10 values of *s*_0_ (0.0001 to 0.5, exponential scale) and 9 values of *ρ* (0 to 0.8, linear scale). Onto the contour map of the simulation results, we place silhouettes of populations from Fig 1 based on their *s*_0_ value and the calculated *ρ* of their environment. The latter is determined from the dominant oceanographic index native to their population region (see Appendix F in S1 File). The greater sensitivity to environmental forcing seen for Type III populations is thus exacerbated by the environment itself, such that regions with more pronounced environmental memory will force even greater variability to these populations.

Environmental autocorrelation accentuates the effects of high mortality in early life stages as predicted by the simple model in Eq (4) and also confirmed by simulations of Model-2 in Fig 4. This figure illustrates how the population variance arising from environmental variability in the mortality rate is boosted by higher correlation in environmental variability. In general higher variability leads to greater extinction rates. Thus increasing fecundity and increasing redness both lead to an increase in extinction rates. However, once the level of autocorrelation or “redness” becomes high (i.e. when the noise behaves in a more non-stationary fashion, typically for values of *ρ*≥0.95) the picture becomes more complicated [37] and forecasts within such regimes are beyond the scope of this study.

Life history strategy determines the effectiveness of conservation actions. Table 1 shows the “conservation gain” resulting from nine different conservation actions for five different species, according to Model-2. We have chosen the magnitudes of the different actions to yield the same gain for the only type-II species in the set, the wandering albatross. We see that for the type-I species (the orca), the most effective action is to increase juvenile survival whereas for the three type-III organisms the most effective action is to reduce mortality in the earliest stage. This shows that the most beneficial conservation actions for type-I populations are not the best for type-III populations. Also, Type I species respond relatively uniformly to different conservation actions. (We have not included in our calculations that Type I organisms having behavioural plasticity might be further buffered against environmental variability.) Differences in conservation outcomes are more pronounced for Type III organisms. Secondly, for the Type III organisms in the table, it is changes in survival early on that can lead to major changes in gain. Simulations also revealed that variance in conservation gain is greater for Type III organisms, so it is more difficult to forecast the degree of success for such populations. Sea turtle populations have been modelled using a variety of techniques [27, 28, 78, 79]. They are long-lived but are most appropriately characterized by Type III strategy and age structure (Fig 1C) as they combine high fecundity, low parental investment, and high first-year mortality. In leatherback turtles, for example, where *s*_0_ = 0.028, juvenile *s*_*k*_ = 0.805, and juvenile stage duration is 15 years [56], the juvenile survival coefficient is 0.039. Thus, hatch-year leatherbacks have greater mortality and variability in hatch-year survival will contribute significantly to overall population variance more so than variability in survival at later life stages.

The results for conservation gain in Table 1 can also be turned around. Although all the interventions of Table 1 are in the form of a boost, we can show exactly analogous behaviour if there are negative impacts, such as pollution or hunting. Type III organisms with high fecundity must also have high mortality rates which by Eq (4) implies greater sensitivity to environmental variations. However, the message of Table 1 is that while Type I organisms (that take a long time to replace themselves) will be sensitive to removal of adults (or nest equivalents), Type III organisms enjoy greater resilience to overharvesting compared to environmental variability. For example, for fungi, which have extreme Type III life cycles, our model predicts that mortality in the adult population (e.g. through harvesting) will have much less effect on future populations than environmental damage that impacts fecundity (or survival to early life stages) (e.g. habitat damage associated with harvesting). Greater sensitivities have been highlighted in bold. It is worth noting that the leatherback sea-turtle has the same pattern as the bass and the conch but is very different from the orca. This shows that in conservation terms, sea turtles may be more suitably identified with type III organisms than with sea-mammals or other type I organisms.

It can be argued that as far as population viability analysis (PVA) is concerned, we do understand the population recovery of Type I organisms. These organisms are sensitive to direct exploitation but resilient to many types of environmental changes. In fact environmental factors are often entirely omitted from PVAs published in conservation journals, considering mainly population dynamics, demographic stochasticity and exploitation mortality. However, the road to recovery for Type III organisms remains mysterious and occluded. It is obvious that Type III organisms pay for any large resilience to harvesting and rapid evolution between generations through a greater vulnerability to environmental variability. Yet, we cannot ignore the dangers of overharvesting even for very high values of fecundity. Numerous conservation failures for Type III organisms show the dangers of betting on high fecundity [42, 44]. Among plants, orchids are Type III strategists. For example, the Lady’s Slipper Orchid (*Cypripedium calceolus*), has a fecundity of 6,000–17,000 seeds. This species was once common in the UK, but it became rare through rapacious collection rather than habitat loss or environmental change [80]. In spite of intense conservation effort for several decades, the remaining population has stubbornly declined to expand. The sustained depression at low populations suffered by such high-fecundity Type III organisms continues to be an unresolved population-dynamics problem as well as a conservation problem [42, 44].

Various metrics have been used to describe the extent to which organisms exhibit Type III life-history, as described in Appendix B (OSM). At present there is no consensus on which of these is likely to be most useful because the issue arises in different areas of applied ecology but only in fisheries science has the issue received much attention. While Type III organisms are in theory just as much the purview of conservation biology, the paradigm of charismatic organisms (mostly Type I) has been hard to shift. However, the universality and dynamic nature of the threat of climate change will require a rethinking of paradigms.

Temporal autocorrelation (i.e. persistence) is a feature of many forms of environmental variability and can have major impacts for extinction predictions [37]. The effect of autocorrelation upon variability is illustrated in Fig 4. Here, we have used Model 2, subject to environmental stochasticity with a fixed variance and a variable level of temporal autocorrelation (AR-1 process). The color codes in Fig 4 are based on the standard deviation of the logarithm of population. Consistent with the foregoing discussion and with Eq (4), population variability increases with increasing autocorrelation (*α*) of the environmental variability. It is worth noting that the largest value *s*_0_ = 0.5 in Fig 4 corresponds to *s*_0_ broadly similar to other values of *s*_*k*_. Note that for *ρ* = 0 (uncorrelated white-noise forcing), the population dynamics are essentially a random walk, so if *ρ*>0, especially close to unity, we find the dynamics have more of a black-noise pattern [81, 82]. Temporal autocorrelation in environmental variability can have various other impacts on population variability, for example through demographic dispersion [35].

Are species inherently fixed in their life history types? Canonical examples of Type I populations (Fig 1) are associated with the traits of low fecundity, strong parental investment, highly evolved social structure, and considerable behavioural complexity. These traits together mitigate the direct impacts of environmental variability to survival. Type III populations, by contrast, without such traits are more easily influenced by environmental variability (Fig 3). However, systemic and persistent pressures could increase mortality at certain life stages, for all life history strategy types, depressing the survival curve from its typical form.

Studies show such negative and chronic population pressures—whether environmental or anthropogenic—may make populations more variable and more susceptible to environmental variability. Under persistent suboptimal environmental conditions, for example, compensation may weaken and we see Type I populations exhibit survival curves more resembling Type II or even Type III populations. This has been separately documented in geographically distinct populations of Steller sea lion (*Eumetopias jubatus*) and the Hawaiian monk seal (*Monachus schauinslandi*) [83, 84]. The wandering albatross which in the early 1990s had a Type II form (Fig 1B), a century earlier perhaps may have more of a Type I population form. Elevated juvenile mortality in this seabird population has been linked in recent years to commercial fisheries [52]. An analysis of ichthyoplankton data from the California Current System showed that fishing increases temporal variability of populations [85], and subsequent analysis found that this was caused by increased instability in population dynamics [1]. In this setting, the challenge to managers would then be to restore natural life history forms (in this case, factors contributing to increasing juvenile survival at-sea) but not to fundamentally alter the native life history patterns themselves. Populations at equilibrium, for example, might also exhibit different patterns than those increasing or decreasing in abundance.

## Conclusions

The survival curve plays a major role in an organism’s sensitivity to climatic variability, especially the distinction between Type I, II and III strategies. It is often supposed that Type I organisms will be most vulnerable to climate change. However, the regime predicted by climate models features not just directional global warming but also increasing climate variance. Our simplified analysis suggests that Type III organisms are likely to be most sensitive to this kind of variability and hence more vulnerable and also that the effectiveness of a specific conservation action depends upon the life-history type. In particular the massive mortalities at early stages intrinsic to Type III species create special sensitivity to environmental variability that is reflected at the population level. Thus, in prioritizing conservation actions, we should treat Type III organisms differently from Type I organisms. While the most effective conservation actions for Type I populations is to avoid killing them, for Type III populations (with high fecundity and low parental investment), conservation goals should focus on protecting early life stages from hostile environments.

## Supporting information

S1 File Development of background theory on stochasticity of survival and fecundity and a more extended discussion and justification of the methods in this study. (DOC)

S2 File Raw data used in this study. (XLS)
