# Supplementary material for: How survival curves affect populations’ vulnerability to climate change
Source: PLoS One. 2018 Sep 6;13(9):e0203124. doi: 10.1371/journal.pone.0203124 (PMC6126862; doi:10.1371/journal.pone.0203124)
Supplement: S1 File — (DOC) [file pone.0203124.s001.doc]

**TITLE:** How survival curves affect populations’ vulnerability to climate change

**AUTHORS:** John M. Halley,^1^ Kyle S. Van Houtan,^2,3^ and Nate Mantua ^4^

**AFFILIATIONS:** ^1^ Department of Biological Applications and Technology, School of Health Sciences, University of Ioannina, 45110, Ioannina, Greece; ^2^ Monterey Bay Aquarium, Monterey, CA 93940, USA; ^3^ Nicholas School of the Environment and Earth Sciences, Box 90328, Duke University, Durham, North Carolina 27708, USA; ^4^ NOAA Fisheries, Southwest Fisheries Science Center, Santa Cruz, California, USA.

**SUPPORTING INFORMATION**

**Appendix A: Theory for Stochasticity of Survival and Fecundity**

The purpose of this section is to show that for organisms with large recruitment mortality and large fecundity (Type-III) environmental stochasticity affects pre-reproductive survival and first-reproduction fecundity in fundamentally different ways.

Figure 2 shows that the population dynamics (in terms of adults) can be simply represented through the replacement coefficient (*R_t_*), which is a product of fecundity (*f_t_*), survival to adulthood (*L_t_*, reproduction) and population size (*N_t_*), all at generation *t*. In general these are time dependent, so:

$N_{t+1}=R_{t}N_{t}=f_{t}\cdot L_{t}\cdot N_{t}$
We will consider both of these separately.

***Survival to adulthood***. Note that there are two timescales. One timescale is *t*=0, *T*, 2*T*, 3*T*,… which represents the discrete breeding seasons. In this model, we assume discrete non-overlapping generations; here the maximum lifetime, *T*, is the same as the generation time and the time to maturity. Then there is the time *k* within one generation, usually measured in days. Suppose, in a population of organisms, at the start of the generation, we begin with a large number of eggs *X*_0_ at time *k*=0 in generation *t*. These will hatch and become juveniles and grow into adults to produce eggs for the next generation, provided they have attained reproductive age at *k=T*. The number of survivors depends on the environment in the following way. Let us define the number of survivors as *X_k_*. Each day there is a proportional loss of individuals subject to a proportional uncertainty depending on the environment that day. An important variable in this theory is *μ_k_* the average mortality rate of recruits (youngest age class), which in the current theory is assumed to remain constant throughout the pre-reproductive period, until time *T*. Thus the proportional loss per day is a random variable with mean *μ_k_* so that we can write:

$X_{k}=[1-\mu_{k-1}-\mu_{k-1}\varepsilon_{k-1}]X_{k-1}$ …(A1)

Here *ε_k_*~N(0,*V_S_*) represents the random proportional change at time *k* to the loss coefficient *μ*_0_ due to environmental perturbations. We can multiply these out as follows:

$$X_{k}=X_{0}\prod_{j=0}^{k-1} \left(1-\mu_{j}-\mu_{j}\varepsilon_{j}\right)\approx X_{0}\exp\left[\sum_{j=0}^{k-1} (-\mu_{j}-\mu_{j}\varepsilon_{j})\right]$$

Note that for small *x* we equated exp(-*x*)≈1‑*x*. Here, we are assuming that daily losses are small. Thus, assuming that the average mortality rate does not change with time and has a value *μ*_0_ and defining the integrated mortality rate to maturity *m_T_* (in this case *m_T_*=*μ*_0_*T*), we find the overall survival to maturity (*L_t_=X_T_/X_0_*) to be:

$L_{t}=\exp\left(-\mu_{0}T-\Delta\mu_{t}\right)=\exp\left(-m_{T}-\Delta\mu_{t}\right)$ …(A2)

Where:

$\Delta\mu_{t}=\mu_{0}\sum_{s=0}^{T-1} \varepsilon_{s}$ …(A3)

The term Δ*μ_t_* may be written as the sum of *T* standard normal variates *ε_s_*~N(0,*V_S_*), one for each day. For large *T* the term Δ*μ_t_* approaches a random Gaussian variable, by the central limit theorem. If the noise processes are standard normal variates, the *ε_s_* are not correlated, the variance of Δ*μ_t_* is *μ*_0_^2^*TV_S_*. If there is autocorrelation in *ε_s_* then we can define the dependence as:

$Var[\Delta\mu_{t}]=\mu_{0}^{2}T\cdot v(T)\cdot V_{S}$ …(A4)

The function *v*(*T*) is a measure of the autocorrelation of environmental variability: it is unity when *ε_s_* is uncorrelated and it increases more strongly with *T* as the autocorrelation in variability increases. It is proportional to *T*^2^ when *ε_s_* is Brownian motion . The function *v*(*T*) will adopt intermediate values for processes with intermediate levels of correlation .

Thus,

Here, V is the symbol for variance and S the symbol for skewness. Skewness thus increases as the attenuation coefficient increases.

At the same time, many will not survive to breed. In fact, if the population is stable in the long term, we expect that the number that survives will produce approximately the same number of eggs as the generation started with.

***Fecundity***. For the females that survive, fecundity also depends on environmental variability. Essentially, females must accumulate bulk and store energy for vitellogenesis. In this case, the equation for energy accumulation is:

$\frac{df}{dt}=(1+\delta_{t})\beta$ …(A6)

In this case *f_t_* represents the total fecundity accumulated to time *t* by the average female while *β* represents the average rate per unit time at which a female can store reproductive potential (such that *f_T_*=*βT*) and *δ_t_* is the environmental variability on this at time *t*. The integration of this equation leads to the following:

$f_{t}=\beta T+\Delta f=f_{T}+\Delta f_{t}$ …(A7)

As before the noise term Δ*f_t_* is given by the sum of environmental random variables and may be written as the sum of *T* standard normal variates *δ_j_*~N(0,*V_f_*), one for each day:

$\Delta f_{t}=\beta\sum_{j=0}^{T-1} \delta_{j}$ …(A8)

In the same way as for Eq. (A4) above, we can derive the variance for Δ*f_t_*:

$Var[\Delta f_{t}]=\beta^{2}T\cdot v(T)\cdot V_{f}$ …(A9)

Here *β* is the daily rate at which egg-mass is accumulated, *T* is the time to maturity and *V_f_* is the variance of the noise as a proportion of *β*.

***Small-noise growth rate***. The overall growth rate can be found from Eq. 3 in the main text as follows:

$r_{t}=\ln\left(\frac{N_{t+1}}{N_{t}}\right)=\ln\left(f_{T}+\Delta f_{t}\right)-m_{T}+\Delta\mu_{t}$ …(A10)

Since we are assuming that in ecological time, the species neither significantly expands nor contracts, then we are assuming that on average each individual reproduces herself. This means that the replacement rate is approximately unity:

$R=f_{T}L=f_{T}e^{-m_{T}}\approx1$ …(A11)

The two sources of variability Δ*f_t_* and Δ*μ_t_* can impact the growth rate (A10) in different ways. In the case where we can apply (A11) and the assumption that Δ*f_t_* <<*f*_T_ and Δ*μ_t_* and *m_T_* <<1 then the growth rate becomes:

$r_{t}\approx\Delta\mu_{t}+\frac{\Delta f_{t}}{f_{T}}$ …(A12)

Assuming Δ*f_t_* and Δ*μ_t_* are independent we can write the variance of *r_t_*,:

$$Var(r_{t})=Var\left(\Delta\mu_{t}\right)+\frac{Var\left(\Delta f_{t}\right)}{f_{T}^{2}}$$

Thus, using (A4), A(9) and (A11) and assuming the autocorrelation structure of both sources of variability are the same:

$Var(r_{t})=\left(V_{f}+m_{T}^{2}V_{S}\right)\frac{v(T)}{T}$ …(A13)

***Large-noise growth rate***. In the case where we cannot assume that variability is small, the situation is much more complicated. Let us consider separately the effects of variability Δ*f_t_* and Δ*μ_t_* on the replacement rate. In the case where Δ*μ_t_*=0 we can write (A10) as:

$$\left.R_{t}\right|_{f}=\left(f_{T}+\Delta f_{t}\right)e^{-m_{T}}=Lf_{T}+L\Delta f_{t}$$

Thus the variance, using (A9) and (A11), is given by:

$\left.Var(R_{t})\right|_{\Delta\mu_{t}=0}=R_{0}^{2}\left(\frac{v(T)}{T}\cdot V_{f}\right)$ …(A14)

Here we also made use of (A7). However, we cannot now assume that *R_0_*≈1 since the mean of *L_t_* for large variation is no longer exp(-*μ*_0_*T*).

If Δ*f_t_*=0 then:

$$\left.R_{t}\right|_{\Delta f_{t}=0}=f_{0}e^{-m_{T}+\Delta\mu_{t}}=R_{0}e^{\Delta\mu_{t}}$$

This is a lognormal random variable LN(0,*σ*^2^), where *σ*^2^=Var(Δ*μ_t_*). Thus, using the fact that the variance of such a variable is [exp(*σ*^2^)-1]exp(*σ*^2^) and also Eq. (A4), we find the variance is:

$$\left.Var(R_{t})\right|_{\Delta f_{t}=0}=R_{0}^{2}\left(e^{\mu_{0}^{2}T\cdot v(T)\cdot V_{S}}-1\right)e^{\mu_{0}^{2}T\cdot v(T)\cdot V_{S}}$$

In the case where the variance is large *σ*^2^=Var(Δ*μ_t_*)>>1 then [exp(*σ*^2^)-1]exp(*σ*^2^)≈exp(2*σ*^2^) so we can write that:

$\left.Var(R_{t})\right|_{\Delta f_{t}=0}\approx R_{0}^{2}\exp\left(2\mu_{0}^{2}T\cdot v(T)\cdot V_{S}\right)=R_{0}^{2}\left(\frac{1}{L_{0}}\right)^{2\mu_{0}\cdot v(T)\cdot V_{S}}$ …(A15)

Thus, as long as *f_T_* and *m_T_* are small, the noise contributions through both fecundity and survival are comparable. However, comparing (A14) and (A15) it is clear that if 2*μ*_0_^2^*Tv*(*T*)*V_S_* is large than the variability acting on survival is going to have a greater affect that that which acts on fecundity. Factors accentuating this asymmetry caused by the exponentiation process are the amplitude and autocorrelation of environmental noise and also *µ*_0_ the mortality rate (and hence the fecundity) as found in Type-III strategies.

***Non-constant Survival***. If mortality rate is not constant, but has different values depending on age for the *T* ages (*μ*_0_, *μ*_1_, *μ*_2_,..., *μ_T_*_‑1_) subject to environmental noise, we have the following modification of (A2)

$$\ln L_{t}\approx-m_{T}-\sum_{k=0}^{s-1} \mu_{k}\varepsilon_{k}$$

In the case of no autocorrelation we can use (A4) with *v*(*T*)=1 to derive the variance of the noise associated with *L_t_*:

$Var(L_{t})\approx V_{S}[\mu_{0}^{2}+\mu_{1}^{2}+\mu_{2}^{2}+...]$ …(A16)

Thus, under conditions of environmental stochasticity classes contribute variability according to the relative sizes of (*μ*_0_^2^, *μ*_1_^2^, *μ*_2_^2^,...). Recalling the observation of Crouse et al (1987) that for sea turtles juvenile was critical, we might wish to ask when a single recruits’ age class could be more important than a protracted stage with several juvenile classes? Let us assume that the overall survival in the first recruit class is the same as for the *T*-1 juvenile classes, so that *μ*_1_=…=*μ_T_*_‑1_=*μ*_0_/(*T*-1). In this case the ratio of these variances will be:

$\frac{V_{R}}{V_{J}}=\frac{\mu_{0}^{2}}{\mu_{1}^{2}+\mu_{2}^{2}+...+\mu_{T-1}^{2}}=\frac{\mu_{0}^{2}}{(T-1)(\mu_{0}/(T-1))^{2}}=T-1$ …(A17)

Thus, even if the overall mortality of the juvenile classes is as large as that of the recruit class, the juvenile mortality creates less population variability because the mortality is spread over more years and much of it gets averaged out.

***Model-2***.

Model 2 is Leslie matrix model with *T*+1 ages in contrast to Model-1 where the population dynamics are free of age structure. In this model *T* is again the maximum longevity but is in general different to the age to maturity and the generation time. The population is the sum of all the age-classes with individual populations *n_k_*:

$$N_{t}=\sum_{k=0}^{T} (n_{k})_{t}$$

Where

$$\left(\begin{matrix} n_{0} \\ n_{1} \\ n_{2} \\ \vdots\\ n_{T-1} \\ n_{T} \end{matrix}\right)_{t+1}=\left(\begin{matrix} f_{0} & f_{1} & f_{2} & \ldots& f_{T-1} & f_{T} \\ s_{0} & 0 & 0 & \ldots& 0 & 0 \\ 0 & s_{1} & 0 & \ldots& 0 & 0 \\ \vdots& \vdots& \vdots& \ddots& \vdots& \vdots\\ 0 & 0 & 0 & \ldots& 0 & 0 \\ 0 & 0 & 0 & \ldots& s_{T-1} & 0 \end{matrix}\right)_{t}\left(\begin{matrix} n_{0} \\ n_{1} \\ n_{2} \\ \vdots\\ n_{T-1} \\ n_{T} \end{matrix}\right)_{t}$$

The survival coefficients are random variables. The average values of the survival coefficients (*s*_0_...*s_T_*_-1_) are the same as measured from real life histories, where *s_k_* is the probability of surviving the *k*^th^ year and getting through to the *k*+1^th^ year. When environmental stochasticity is present, each parameter is affected equally. Noise enters the population dynamics through the extinction exponent, so that each survival term *s_k_* is multiplied by a random factor and takes the form

$$s_{kt}=\exp[-\mu_{k}-\mu_{k}\varepsilon_{t}]=s_{k}\exp[-\mu_{k}\varepsilon_{t}], 0\leq k\leq K$$

where *ε_t_* is a Gaussian autoregressive term (first-order), namely

$$\varepsilon_{t+1}=\rho\varepsilon_{t}+\delta_{t}, \delta_{t}\sim N(0,\sigma^{2})$$

The first age of reproduction *k*_min_ and the last age of reproduction *k*_max_ are based on real measurements but we assume that average fecundity *f*_0_ is the same for all reproductive ages. Thus:

$$\begin{matrix} f_{kt}=f_{0}+\Delta f_{t} \forall k_{\min}\leq k\leq k_{\max} \\ =0 \text{otherwise}. \end{matrix}$$

The precise value of average fecundity is chosen so that the overall average growth rate is zero, the same approach as adopted by Essington et al. . This can be done by using the Euler-Lotka equation:

$\sum_{k=0}^{K} e^{rk}l_{k}f_{k}=1$ …(A18)

We fix at zero the linear growth rate *r*. Also, in Model-2 the fecundity of all active classes is assumed to be the same, so *f_k_=f*_0_ for all *k* in the range *k*_min_, *k*_max_. This leads to the requirement that

$f_{0}=\frac{1}{\sum_{k=k_{\min}}^{k_{\max}} l_{k}}$ …(A19)

Model 2 is a more realistic than Model 1, as it contains the observed age-structure, for which the parameters better characterize real organisms, although it still involves some simplifying assumptions, since we ignore density dependence and assume all age classes experience the same proportional perturbation in a given year.

**Appendix B: Measures of Life Strategy**

Here we explain the steps to establish a measure of life strategy.

**S1 Figure. Three different life-history types as a function of relative age, showing an extreme example of Type I** (dotted line).

Relative age *u* is defined as the age, *k*, relative to the lifespan *T*, that is *u=k/T*, so the scale is [0,1]. The relative abundance is expressed on a log scale. Suppose we have a cohort of organisms beginning with *n*_0_. By the start of the second age-class we have *n*_1_=*s*_0_*n*_0_ in the cohort, by the third it is *n*_2_=*s*_1_*s*_0_*n*_0_ and so on. By the end of the last stage (relative age unity) the last individual is ready to die, so the number left is unity. The logarithm of this goes from ln(*n*_0_) to zero. We have scaled the axis so z(*k*)=ln(*n_k_*)/ln(*n*_0_), so that the scale is the same for all organisms. Thus all life strategies’ survival curves are contained within the unit square [0,1]× [0,1]. If the survival curve lies above the diagonal then the strategy is Type I; if it is below it the strategy is type III; Type II strategies lie exactly upon the diagonal.

We define the strategy by a number *A*, which is twice the area between the survival curve and the diagonal *z*_2_(*u*) that joins (0,1) and (1,0).

$A=2\int_{0}^{1} \left[z(u)-z_{2}(u)\right]du=2\int_{0}^{1} \left[z(u)-(1-u)\right]du=2\int_{0}^{1} \left[z(u)+u\right]du-2$ …(B1)

Clearly, the most extreme Type-I strategy is one that approaches the boundary of the square (as shown in Figure B1). The area between this curve and the diagonal approaches one half, hence *A* is just short of unity. For type-III strategies *A* is negative and for Type-II strategies it is zero. Thus:

0<*A*<+1 for Type-I

*A≈*0 for Type-II ...(B2)

-1<*A*<0 for Type-III

In using this metric, we must also decide how close to *A*=0 constitutes an organism with a Type-II strategy. In the text we use the range |*A*|≤0.21, although the Wandering Albatross should be counted in this scheme as Type I.

Clearly, not all organisms may be neatly categorized into this structure. However, this framework provides a simple metric to quantify the life history differences we have considered in this paper.

**Odds Ratio**

A second method we describe as 1/*S_M_*, the inverse survival to maturity, also sometimes called the Odds Ratio of an individual reaching sexual maturity. 1/*S_M_* is the inverse of the proportion of recruits that reach the reproductive phase. It functions as a simple index of life history strategy or type (Figure 1), as it reflects the total accumulation of tradeoffs in fecundity, parental investment, age at maturity, and other factors that ultimately impact survival to reproductive age. For African elephants who are type I, 1/*S_M_* equals 1.3 and the probability of each newborn surviving to breeding age is 0.769. For Pacific leatherback sea turtles who are type III, 1/*S_M_* equals 1,277 and the individual probability of reaching breeding age is < 0.0008. For a Michigan painted turtle which is in the middle type II, 1/*S_M_* equals 10, and the individual probability of reaching breeding age is 0.10. Table S1 provides more details.

**Steepness**

Fisheries science commonly uses a parameter called steepness. This is deﬁned, for a given density-dependent recruitment model, as the fraction of recruitment from an unﬁshed population obtained when the spawning stock biomass is 20% of its unﬁshed level. So, a value of steepness close to 0.2 implies that the population is only replacing itself. A value of 0 means extinction. On the other hand, if it is close to unity then the population is virtually immune to harvesting pressure. Organisms with large values of steepness must have large values of fecundity. As well as being a way of measuring the resilience to overfishing, steepness is also considered as a measure of environmental control of the stock. When steepness is unity then recruitment is often considered to be “environmentally driven.” Mangel et al. have pointed out that this is biologically inconsistent. If an organism is strongly affected by environmental forcing, this will generate a spectrum of steepness rather than any single value.

So if recruitment has a value *Y(N)=Y*_0_ when *N=N*_0_, that is *Y*_0_=*Y*(*N*_0_) then the steepness *h* satisfies the following equation :

$hY_{0}=Y(0.2N_{0})$ …(B3)

So that:

$h=\frac{Y(0.2N_{0})}{Y(N_{0})}$ …(B4)

Typically, *Y*(*N*) is a nonlinear density dependent function. As a result, we do not use steepness in this paper because it is associated with density-dependent models.

**Appendix C: Further Data for Populations in Fig 1 and Simulations in Table 1**


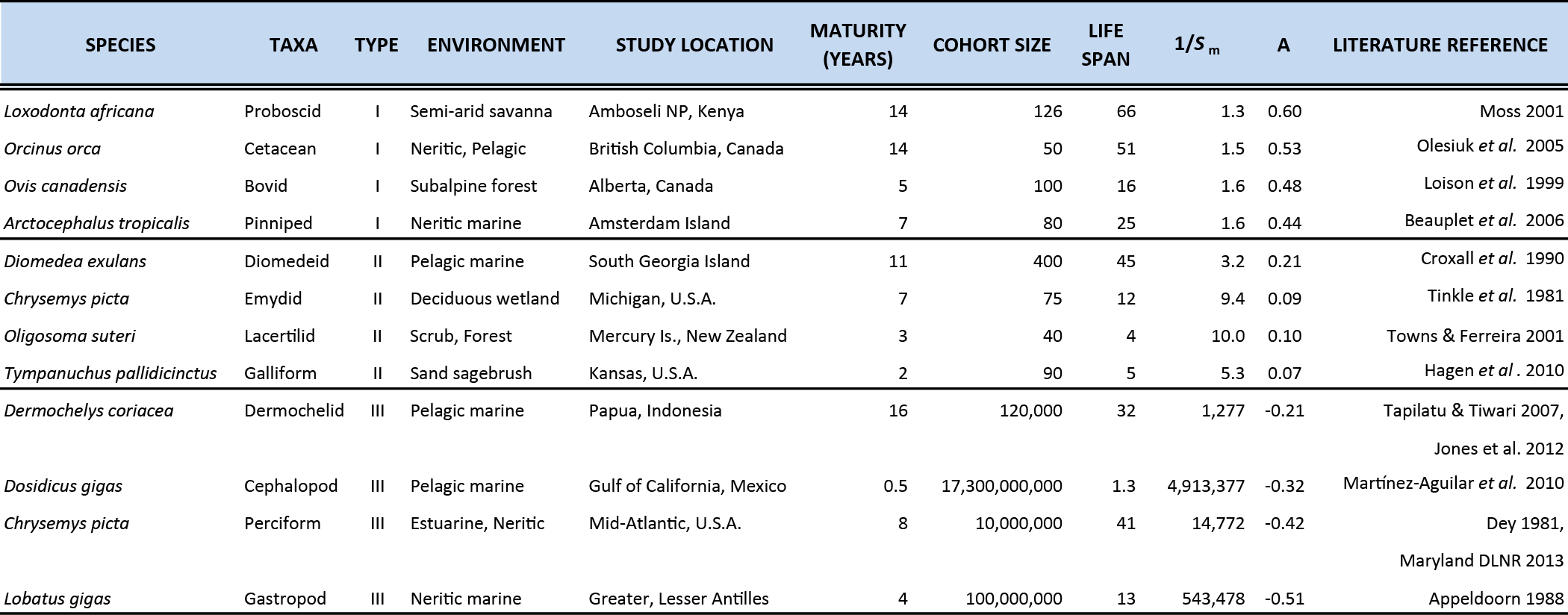


**S1 Table. Further data for populations in Fig 1 and Table 1.** The following table gives some parameters relevant to the organisms in Fig 1. “Type” is life history strategy, “Maturity” is the age in years in which breeding begins, “Cohort size” is *n*_0_ or the annual population production, “Life span” is the time in years where cohort size falls to unity, “1/*S*_m_” is the odds ratio (inverse probability) of an individual reaching sexual maturity, and “*A*” is the life history strategy quantity (see OSI, Section B). Order from top to bottom is the same as in the main text, Figure 1. Full citations for references are in the in main text.

**Parameterization and Simulations**

In all work involving Model-2, we used the following steps to parameterize the model. The references in Table S1 provide cohort sizes, ages of maturity and longevity. The survival coefficients, *s_k_*, were estimated based on the cohort sizes *n_k_*, according to the equation *s_k_*=(n_k_‑*n_k_*_+1_)/n_k_. Fecundities were assumed to be equal for all reproductive classes, *f_k_*=*b*. If no specific post-reproductive age was given, we assumed that reproduction could happen until the last age class. To get *b*, we solved Eq.(6) for the value of *b* that gives an overall population growth rate of zero in the absence of stochasticity.

In simulations, for each year *t*, a Gaussian random variable N(0,*σ*^2^) was generated to represent the environment each year. This was in general autocorrelated according to equation [8] but for Table 1 it is zero (*ρ*=0). This gives *ε_t_* for this year. For each age class, this number is multiplied by the corresponding mortality rate and exponentiated according to Eq. [7] to find *s_kt_*.

$$s_{kt}=\exp[-\mu_{k}-\mu_{k}\varepsilon_{t}]$$

We assumed a standard deviation of the environmental parameters was *σ*=0.1 (see Eq. 7). This gives St.Dev[ln*N_t_*]=0.058 in the absence of any conservation intervention

We assume the same environmental process affects fecundity *f_kt_* as affects survival, so that Eq.(9) becomes:

$f_{kt}=f_{0}+\Delta f_{t}=f_{0}+f_{0}q\varepsilon_{t} \forall k_{\min}\leq k\leq k$ …(C1)

Thus, a large value of *ε_t_* representing a “friendly” environment, in this simulation, acts both the decrease mortality in all age classes and to increase fecundity by the same amount in all reproductive classes. However, we do not expect the environment to affect fecundity exactly the way it affects mortality rate, which is the reason for the scaling factor *q*. We choose *q* so that the standard deviation of environmental variability is in the case of the albatross (*Diomedea exulans*) is the same for fecundity variability acting alone as it is for survival variability acting alone. This leads to a value of *q*=1.55, which we use throughout. The assumptions *σ*=0.1 and *q*=1.55 allow us to explore the importance of changing the reproductive strategy from type-I through to type-III. The simulations in this study do not introduce randomness in any other way, only through *ε_t_*.

**Appendix D: Populations Showing Evidence of Change of Strategy Type**

In declining populations, persistent suboptimal environmental constraints can decrease juvenile survival, delay maturity (relative to total life span), and give the appearance that preferentially Type I populations have a lower life history strategy (i.e. Type II or III).

S2 Fig has the same form as Figure 1 in the main text, but compares within-species variations at the population level for two species of pinnipeds. Although both species are considered “endangered” under the U.S. Endangered Species Act the population dynamics are very different. Persistent suboptimal environmental conditions have resulted in the Marmot (western) Steller Sea Lion population having a much lower survival to maturity, and the appearance of being a Type II population, in comparison with the Graves Rock (eastern) population. The grey line represents the Marmot (western) population that is in chronic decline, while the black line describes the cohort survival for Graves Rock (eastern) population that has increasing abundance . For Hawaiian monk seals, similar constraints have resulted in the French Frigate Shoals (Northwestern Hawaiian Islands) population also having a significantly reduced survival to maturity and the appearance of being a Type II population, by comparison to the Main Hawaiian Islands population group . Both species have evolved Type I life history strategies, similar to the species listed described in Figure 1 and Table S1, and will evince population declines when persistent environmental conditions force unsustainably low juvenile survival. Full citations for references are available in the OSI.

**S2 Table. Metadata and literature sources for the species and populations considered in S2 Figure**. “Type” is life history strategy, “Maturity” is the age in years in which breeding begins, “Cohort size” is *n*_0_ or the annual population production, “Life span” is the time *t* in years where *n*_t_ < 1, “1/*S*_m_” is the odds ratio of a individual reaching sexual maturity, and “A” is the life history strategy quantity (Appendix B in S1 File ). For Location, “MHI” is the Main Hawaiian Islands, and “FFS, NWHI” is French Frigate Shoals in the Northwestern Hawaiian Islands. Referenced studies provide background and underlying parameters .

**
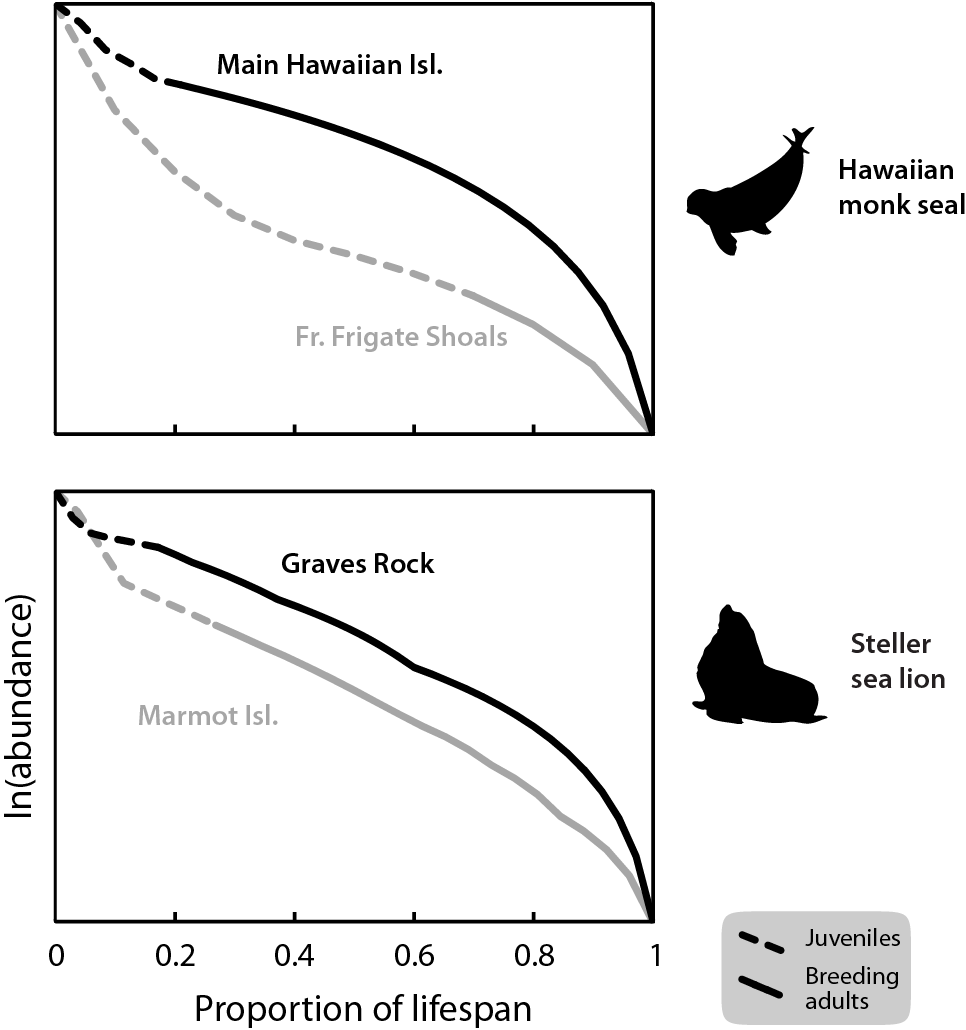
**

**S2 Figure. Life history type variability within species.** Dotted lines represent juvenile stages, solid lines begin at the earliest onset of breeding. Both abundance and lifespan proportion are scale normalized at the population level. Full metadata and literature references available in Appendix F S2 Table, preceding this figure.

**Appendix E: Relation to Previous Sea Turtle Studies**

Marine turtles are characterized by Type III age structure. This implies that marine turtles are highly susceptible to disturbances in their environment, and their populations are strongly affected by environmental variation. Thus, the primary determinant of adult population density for marine turtles is likely to be environmental conditions in their birth. This conclusion seems rather different to that of previous studies who claim that large juveniles and adults, respectively, are the most pivotal groups. Crouse et al. for example, argued that if an initial population of 500,000 animals is subjected to a 50% reduction in large juvenile survival, breeding females will be extinct in 40 years. However, if the same 50% reduction occurs in the first year class, the same population retains > 52 adult females in that time. Thus, they concluded large juveniles are more consequential to the population’s persistence. However, the impact of each life stage depends on its overall survival coefficient, which is correlated with stage duration. Thus, “large juveniles” is not a single year class, but the complete demographic stage that spans 8 years and has a much lower survival coefficient (0.044) than that of the first year of life (*s*_0_=0.675). In practical terms, this means that a conservation measure involving large juveniles should be imposed for the entire 8 years in order to have the said effect.

Generally, s_0_ has been recognized as a difficult parameter to measure. The value of *s*_0_=0.675 used by Crouse et al. , more typical for species of Type I like mammals or birds (See Figure 1, S1 Table), seemed high even at the time and was subsequently reduced to 0.370 by a team from the same research group , which is exactly the value of s_0_ for Kemp’s Ridley sea turtles (*Lepidochelys kempii*) in . This highlights the logistical problems in estimating values for *s*_0_. More recent studies of marine turtles using added empirical monitoring and improved technologies arrive at much lower values, such as *s*_0_=0.028 for the leatherbacks . If s_0_ for loggerheads is this low then recruitment mortality has a greater effect than even the 8 large-juvenile years together. Figure S3 shows Figure 4 with the loggerhead included. In it we use the two sets of parameters generating two points separated by an arrow. One point (P) is associated with the parameters of Crouse et al. , which generates relatively little sensitivity to environmental variability. For the other point (Q) we have made recruit survival close to that of the leatherback. This dramatically increases the modeled relative population variance and has important implications for loggerhead populations’ long-term population dynamics, and vulnerability to climatic forcing and climatic change. Thus, the differences from earlier studies regarding our recommendations, hang mainly upon the values assumed for *s*_0_.


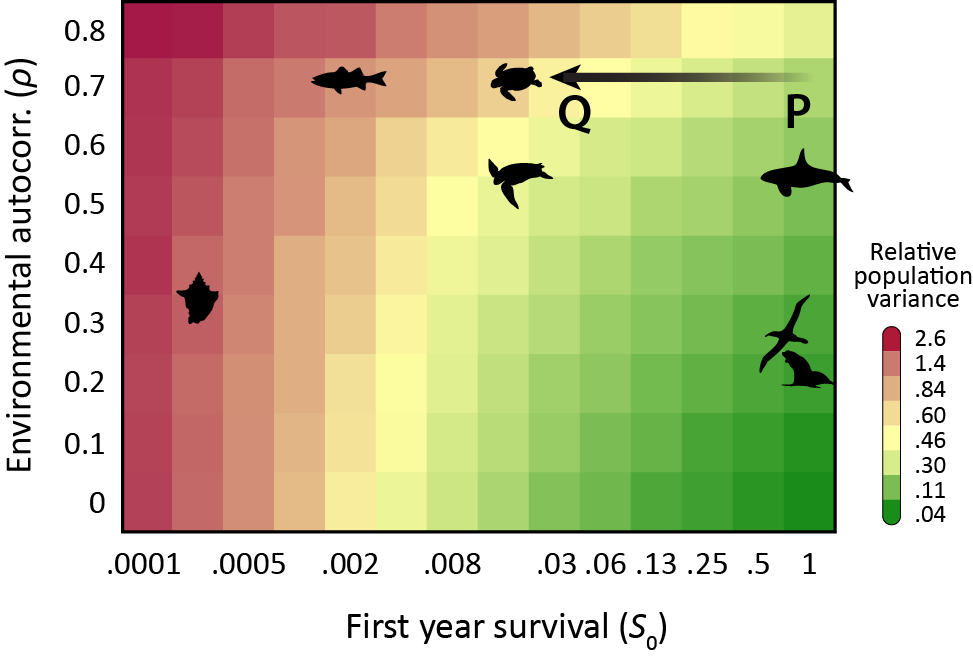


**S3 Figure. The combined influence of early life stage survival and environmental autocorrelation, including loggerhead sea turtles**. This is an expansion of Figure 4 from the main text to emphasize the importance of life history type, and in particular the importance of accurate estimation of *s*_0_. In addition to the populations shown in Figure 1, we add here loggerhead sea turtles using varying values of *s*_0_ from 0.675 (highlighted by “P”) to 0.020 (highlighted by “Q”). The former value is from Crouse et al. 1987 with the latter close to that of Jones et al. 2012.

**Appendix F: Environmental Autocorrelation Series**

**S3 Table. Locations, environmental time series, and rho values for six marine taxa.** This table represents the full metadata used in the main text Figure 4, including the environmental indices and their calculated autocorrelation (rho). All environmental data are from the NOAA Earth System Research Laboratory, Physical Sciences Division, and are freely available at <http://esrl.noaa.gov/psd/data/climateindices/list/>. “*S*_0_” is the annual survival in the first year and “Environ. Time Series” is the dominant environmental index for the location where the population occurs. In order of appearance, here “PDO” is the Pacific Decadal Oscillation, “AAO” is the Antarctic Oscillation, “AMO” is the Atlantic Multidecadal Oscillation, and “NTA” is the North Tropical Atlantic SST Index. “SST derived” indicates that an SST-based index (PDO, AMO, etc.) was unavailable and therefore we determined from the one year lag autocorrelation of the annual SST averages. An extended discussion of the AMO and its ecological impacts is provided by Nye et al. . Listed here but not pictured in Figure 4 is the Subantarctic fur seal; its silhouette would be essentially on top of the (pictured) wandering albatross.

**References**

1. Halley JM. How do Scale and Sampling Resolution Affect Perceived Ecological Variability and Redness? In: Vasseur DA, McCann KS, editors. The Impact of Environmental Variability on Ecological Systems. Dordrecht: Springer Netherlands; 2007. p. 17-40.

2. Halley JM, Inchausti P. The increasing importance of 1/f-noises as models of ecological variability. Fluctuation and Noise Letters. 2004;04(02):R1-R26. doi: 10.1142/s0219477504001884.

3. Essington TE, Ciannelli L, Heppell SS, Levin PS, McClanahan TR, Micheli F, et al. Empiricism and Modeling for Marine Fisheries: Advancing an Interdisciplinary Science. Ecosystems. 2017;20(2):237-44. doi: 10.1007/s10021-016-0073-0.

4. Mangel M, MacCall AD, Brodziak J, Dick E, Forrest RE, Pourzand R, et al. A perspective on steepness, reference points, and stock assessment. Can J Fish Aquat Sci. 2013;70(6):930-40.

5. Mangel M, Brodziak J, DiNardo G. Reproductive ecology and scientific inference of steepness: a fundamental metric of population dynamics and strategic fisheries management. Fish Fish. 2010;11(1):89-104.

6. Hastings KK, Jemison LA, Gelatt TS, Laake JL, Pendleton GW, King JC, et al. Cohort effects and spatial variation in age-specific survival of Steller sea lions from southeastern Alaska. Ecosphere. 2011;2(10):1-21. doi: 10.1890/ES11-00215.1.

7. Pendleton GW, Pitcher KW, Fritz LW, York AE, Raum-Suryan KL, Loughlin TR, et al. Survival of Steller sea lions in Alaska: a comparison of increasing and decreasing populations. Can J Zool. 2006;84(8):1163-72.

8. Harting Jr. AL. Stochastic simulation model for the Hawaiian monk seal. Bozeman, MT: Montana State University; 2002.

9. HMSRP. Population Summary for NWHI Monk Seals in, Internal Report IR-14-011. Honolulu, HI USA: NOAA Fisheries, Pacific Islands Science Center; 2014 12 January 2007. 27 p.

10. HMSRP. 2013 MHI Hawaiian Monk Seal Population Summary, Internal Report IR-14-013. Honolulu, HI USA: NOAA Fisheries, Pacific Islands Science Center; 2014 12 January 2007. 7 p.

11. Crouse DT, Crowder LB, Caswell H. A Stage-Based Population Model for Loggerhead Sea Turtles and Implications for Conservation. Ecology. 1987;68(5):1412-23. doi: 10.2307/1939225.

12. Arendt MD, Schwenter JA, Witherington BE, Meylan AB, Saba VS. Historical versus Contemporary Climate Forcing on the Annual Nesting Variability of Loggerhead Sea Turtles in the Northwest Atlantic Ocean. PLoS ONE. 2013;8(12):e81097. doi: 10.1371/journal.pone.0081097.

13. Heppell SS, Crouse DT, Crowder LB, Epperly SP, Gabriel W, Henwood T, et al. A population model to estimate recovery time, population size, and management impacts on Kemp’s ridley sea turtles. Chel Conserv Biol. 2005;4(4):767-73.

14. Jones TT, Bostrom BL, Hastings MD, Van Houtan KS, Pauly D, Jones DR. Resource Requirements of the Pacific Leatherback Turtle Population. PLoS ONE. 2012;7(10):e45447.

15. Nye JA, Baker M, Bell R, Kenny A, Kilbourne KH, Friedland KD, et al. Ecosystem effects of the Atlantic Multidecadal Oscillation. J Mar Syst. 2013;133(2014):103-16. doi: 10.1016/j.jmarsys.2013.02.006.
